# 826. Acellular-fish-xenograft as a Salvage Device for Severe Burns and Wounds Following Failure of Alternative Therapies

**DOI:** 10.1093/jbcr/irag033.265

**Published:** 2026-04-06

**Authors:** Paige Richards, Hannah L Moore, Alyssa D Brown, Alan Pang

**Affiliations:** Texas Tech University Health Sciences Center, Lubbock, TX; Texas Tech University Health Sciences Center, Lubbock, TX; Texas Tech University Health Sciences Center, Lubbock, TX; Texas Tech University Health Sciences Center, Lubbock, TX

## Abstract

**Introduction:**

Severe burns and complex wounds are challenging in nature to manage due to extensive tissue damage, high infection risk, and the frequent involvement of critical underlying structures. Standard treatment involves early excision and definitive coverage, typically utilizing split-thickness skin grafting (STSG). However, successful grafting requires a well-vascularized wound bed, which may be compromised in patients with large total body surface area (TBSA) involvement or with comorbidities. When wounds are not immediately graftable, additional therapies such as cadaveric allografts, biodegradable temporizing matrix, autologous skin cell suspension, and negative pressure wound therapy are employed to further promote granulation and prepare the wound bed. Despite these interventions, some wounds remain refractory to healing. In such cases, alternative biologic substrates like an acellular fish xenograph, have shown promising potential in offering a biologically innovative alternative for complex wound healing. It's structurally similarity to human dermis and allows for the promotion of angiogenesis, reduction of inflammation, and acceleration of granulation. Acellular fish xenograft shows promising potential as a salvage therapy in patients who fail standard wound coverage modalities. This case series is designed to assess the efficacy of acellular fish xenograft as a salvage therapy in complex wound or burn cases where initial biologic or synthetic wound coverage products failed.

**Methods:**

A retrospective chart review was conducted on seven patients treated at our hospital between July 2023 and December 2025. All patients had prior failed wound coverage using standard products, defined as <80% graft take or absent granulation. Data included demographics, comorbidities, burn severity, BMI, and timing metrics. Median time from wound to acellular fish xenograft application was 30 days (range 16–50). Outcomes included graft take and final wound closure following acellular fish xenograft.

**Results:**

The cohort (n = 7) had a mean age of 48 years (range 28–86). All seven had extensive prior interventions; acellular fish xenograft was used as a last-resort adjunct. Following acellular fish xenograft application, 5 of 7 patients achieved >80% graft take, with an average healing time of 10.4 days to STSG. No adverse events were attributed to acellular fish xenograft use.

**Conclusions:**

Acellular fish xenograft proved to be effective as a salvage skin substitute in 72% of patients with refractory burn and wound beds where traditional therapies had failed. This preliminary data supports its consideration for use earlier in the treatment course for patients with anticipated poor healing.

**Applicability of Research to Practice:**

Acellular fish xenograft proved to be effective as a salvage skin substitute in 72% of patients with refractory burn and wound beds where traditional therapies had failed.

**Funding for the study:**

N/A.

